# The co‐design of a Brain Health Game for children to reduce risk of dementia

**DOI:** 10.1002/alz.085330

**Published:** 2025-01-09

**Authors:** Christine Brown Wilson, Rebecca Townsend, Gary Mitchell, Stephanie Craig, Gillian Carter, Patrick Stark, Sonya Clarke, Susie Wilkie, Claire T McEvoy

**Affiliations:** ^1^ School of Nursing & Midwifery, Queen’s University Belfast, Belfast, Northern Ireland United Kingdom; ^2^ School of Biomedical, Nutritional and Sport Sciences/ Newcastle University, Newcastle upon Tyne, England United Kingdom; ^3^ School of Nursing & Midwifery, Queens University Belfast, Belfast United Kingdom; ^4^ School of Nursing & Midwifery, Queens University Belfast, Belfast, Northern Ireland United Kingdom; ^5^ Centre for Public Health, Queen’s University Belfast, Belfast United Kingdom

## Abstract

**Background:**

Dementia prevalence is projected to treble worldwide by 2040 highlighting the critical need for effective primary dementia prevention strategies. Initiatives to shape health behaviours/beliefs in childhood increase likelihood of engagement in healthy lifestyle behaviours during adulthood however some subgroups of children are more vulnerable to poor brain health during ageing. Therefore, children must have opportunities to learn about how they can engage in behaviours to improve cardiovascular and psychological health that will protect their brain as they age, contributing to dementia risk reduction (DRR) in later life. Serious digital games (SDG) are fun, interactive and engaging, having been used successfully for education and behaviour change. No SDGs to promote brain health/DRR for children currently exist.

**Method:**

A SDG, *‘my Superbrain’* was codesigned with primary school children in Northern Ireland. The aims of this study were: to examine changes in understanding brain health/DRR via pre/post‐test evaluation of the SDG among children aged 8‐11 years old and determine the acceptability and feasibility of a novel SDG for DRR in children. Children completed the Kids Brain Health Questionnaire pre/post playing the Game with a modified System Usability Scale (SUS). All data analyses were conducted in SPSS version 29 following matching of pre/post data. Due to non‐normally distributed data, the non‐parametric Wilcoxon signed‐rank test was used to examine the change from pre‐test to post‐test scores on the Kids Brain Health Questionnaire.

**Result:**

The SDG was evaluated with 46 participants. Most participants reported not having a family member/close friend living with dementia (71.7%). A Wilcoxon signed‐rank test showed a statistically significant median increase (*Mdn* = 1.00) in Kids Brain Health Questionnaire scores from pre‐test levels (*Mdn* = 52.00) to post‐test levels (*Mdn* = 53.0), *z* = 2.90, *p* = .004. A mean score of 36.15 out of a possible 50 was found for the SUS.

**Conclusion:**

The findings demonstrate ‘evidence of promise’ for early intervention stage testing with a small but statistically significant increase in knowledge for children after using the SDG, *‘my Superbrain’*. Participants also found the game acceptable, suggesting further work is warranted to test the SDG on a larger sample of children.

